# Modulation of Inflammation and Gut Microbiota by a *Bifidobacterium longum* Extracellular Vesicle-Based Drug Delivery System for Alleviating Inflammatory Bowel Disease

**DOI:** 10.3390/pharmaceutics18050553

**Published:** 2026-04-30

**Authors:** Chunlei Ma, Shang Shi, Wenke Wang, Boqing Li, Zhiqin Li, Yingzi Cui, Fangshu Li, Xiaoying Chen, Ying Zhang

**Affiliations:** 1 School of Basic Medical Sciences, Binzhou Medical University, 346# Guanhai Road, Yantai 264003, China; machunlei0305@163.com (C.M.); sshi0811@163.com (S.S.); 17854317060@163.com (W.W.); lizhiqin1632023@163.com (Z.L.); m17865587272@163.com (Y.C.); 18854797639@163.com (F.L.); 18111580490@163.com (X.C.); 2 Department of Microbiology, College of Life Sciences, Shandong Agricultural University, Taian 271018, China

**Keywords:** inflammatory bowel disease, *Bifidobacterium longum*, extracellular vesicles, gut microbiota, inflammatory response, colonic injury

## Abstract

**Purpose**: Inflammatory bowel disease (IBD) is a chronic inflammatory disorder strongly associated with intestinal microbial dysregulation. Although 5-aminosalicylic acid (5-ASA) is widely used in the clinical management of IBD, its therapeutic efficacy is often limited. To address this, the present study aimed to develop a bifidobacterium-derived extracellular vesicle-based drug delivery system (B-MVs@5-ASA) to enhance the therapeutic outcomes of IBD. **Methods**: B-MVs were isolated by PEG precipitation and loaded with 5-ASA via sonication to obtain B-MVs@5-ASA. Their morphology, particle size, zeta potential, and encapsulation efficiency were analyzed using TEM, DLS, and UV spectrophotometry. Cellular uptake, cytotoxicity (LDH and NO assays), and anti-inflammatory effects were assessed in RAW 264.7 and Caco-2 cells. A DSS-induced colitis mouse model was established to evaluate therapeutic efficacy. Cytokines (ELISA), colon histopathology (H&E), tight-junction proteins (IF), and gut microbiota composition (16S rRNA sequencing) were systematically analyzed. **Results**: B-MVs@5-ASA exhibited a particle size of 104.3 ± 2.81 nm and an encapsulation efficiency of 11.14% ± 3.63%. B-MVs@5-ASA exhibited the strongest anti-inflammatory effect in vitro and most effectively alleviated DSS-induced colitis in vivo, outperforming monotherapies in reducing inflammation, tissue damage, and enhancing barrier integrity. B-MVs@5-ASA further promoted goblet cell regeneration and beneficially modulated the gut microbiota by enriching *Akkermansia* and suppressing *Escherichia*, thereby restoring microbial homeostasis. **Conclusions**: B-MVs@5-ASA provides potent anti-inflammatory and mucosal-protective effects by modulating cytokine balance, enhancing epithelial barrier function, and reshaping gut microbiota. These findings highlight probiotic vesicle-based nanoplatforms as a safe and promising strategy for targeted IBD therapy.

## 1. Introduction

Inflammatory bowel disease (IBD), including Crohn’s disease (CD) and ulcerative colitis (UC), is a chronic inflammatory disorder characterized by persistent mucosal injury, epithelial barrier disruption, and gut microbiota dysbiosis [1,2]. Hallmarks of disease progression include tight junction loss, overgrowth of pathogenic bacteria, and reduced microbial diversity, ultimately impairing mucosal homeostasis. Current clinical treatments, such as 5-aminosalicylic acid (5-ASA), corticosteroids, antibiotics, and immunosuppressants, can alleviate symptoms but are limited by incomplete therapeutic efficacy, systemic toxicity, and high treatment burden [3]. Therefore, the development of safe, economical, and effective therapeutic strategies remains an urgent need.

Bacterial extracellular vesicles (bEVs) are nano-sized membrane vesicles released through membrane budding and have recently gained attention as bioactive nanocarriers [4]. Depending on the bacterial source, they are categorized as outer membrane vesicles (OMVs) in Gram-negative bacteria and membrane vesicles (MVs) in Gram-positive bacteria [5]. Probiotic-derived bEVs can deliver functional biomolecules that enhance mucus secretion, reinforce epithelial barrier integrity, attenuate oxidative stress, and modulate inflammatory signaling [6,7,8]. EVs from beneficial gut bacteria, including *Bifidobacterium longum*, *Clostridium butyricum*, and *Akkermansia muciniphila*, have demonstrated therapeutic potential in experimental colitis models [9,10,11]. Among these probiotic vesicles, *B. longum*-derived membrane vesicles (B-MVs) are particularly attractive owing to their natural stability, biocompatibility, mucus-penetrating capability, and inherent intestinal targeting [12]. Their bilayer architecture enables efficient loading of both hydrophilic and hydrophobic molecules, and their inflammation-tropic properties facilitate preferential accumulation at diseased sites.

In this study, we designed a *B. longum* membrane vesicle-based 5-ASA delivery system (B-MVs@5-ASA) to combine the intrinsic bioactivity of probiotic-derived vesicles with the established anti-inflammatory efficacy of 5-ASA. Compared with conventional probiotic supplementation, B-MVs provide a cell-free nanoplatform with improved stability, biosafety, and mucus-penetrating potential, while also avoiding the variability associated with live bacterial survival and colonization. We therefore hypothesized that loading 5-ASA into B-MVs could achieve a more efficient local anti-inflammatory effect through the combined actions of the vesicle carrier and the encapsulated drug. Based on this rationale, we systematically assessed its physicochemical properties, biocompatibility, and immunomodulatory activity in vitro, and further evaluated its therapeutic efficacy in a dextran sulfate sodium (DSS)-induced colitis mouse model. In addition, we examined the role of B-MVs@5-ASA in reshaping gut microbiota composition, regulating inflammatory cytokine profiles, and restoring intestinal barrier integrity. Collectively, this work provides new mechanistic and experimental evidence supporting probiotic vesicle-based microecological interventions for the treatment of inflammatory bowel disease.

## 2. Materials and Methods

### 2.1. Culture of Bacteria and Cells

*B. longum* (strain BNCC 185354) was purchased from BeNa Culture Collection (Beijing, China). The bacteria were cultured anaerobically at 37 °C in an anaerobic workstation (atmosphere: 85% N_2_, 10% CO_2_, 5% H_2_) using MRS broth supplemented with 0.5 g/L L-cysteine. Murine macrophage RAW 264.7 cells (Cellosaurus: CVCL_0493) were preserved by the Department of Pathogen Biology, Binzhou Medical University. Human colorectal adenocarcinoma Caco-2 cells (Lot No. CL-0050; Cellosaurus: CVCL_0025) were obtained from Wuhan Procell Life Science & Technology Co., Ltd. (Wuhan, China). Both cell lines were maintained in a complete culture medium (composed of 89% DMEM, 10% fetal bovine serum, and 1% penicillin-streptomycin) at 37 °C in a 5% CO_2_ incubator.

### 2.2. Preparation of B-MVs

Extracellular vesicles from *B. longum* (B-MVs) were isolated using polyethylene glycol (PEG) precipitation. Briefly, *B. longum* was cultured anaerobically in MRS broth at 37 °C for 48 h, after which the culture was centrifuged at 6000× *g* for 5 min to remove bacterial cells. The supernatant was further clarified by centrifugation at 8000× *g* for 10 min (three times) and filtered through a 0.22 μm membrane. A 40% (*w*/*v*) PEG 8000 solution was added to the filtrate at a ratio of 7.5 mL to 30 mL, mixed, and incubated overnight at 4 °C. The precipitated vesicles were collected by centrifugation at 16,000 × g for 1 h at 4 °C and resuspended in 300 μL of sterile PBS. Samples were aliquoted and stored at −80 °C until use.

### 2.3. Determination of the 5-ASA Encapsulation Efficiency

Standard solutions of 5-ASA were prepared at concentrations of 5, 10, 20, 30, 40, 50, and 60 μg/mL. The maximum absorption peak was identified at 300 nm using UV spectroscopy, and a standard curve was constructed from the UV absorption spectra of these solutions. For the encapsulation efficiency (EE) assay, 5-ASA solution was mixed with B-MVs suspension at a volume ratio of 2:1 (5-ASA:B-MVs), followed by sonication and incubation for 2 h. The mixture was then centrifuged, and the supernatant containing unencapsulated 5-ASA was collected. The EE was calculated in triplicate using the formula: EE (%) = [(Total amount of 5-ASA − Amount of free 5-ASA)/Total amount of 5-ASA] × 100%, based on UV measurements.

### 2.4. Preparation and Characterization of B-MVs and B-MVs@5-ASA

B-MVs@5-ASA was prepared by sonicating 5-ASA solution (1 mg/mL) with B-MVs suspension (5 mg/mL) at a volume ratio of 2:1 (5-ASA:B-MVs, *v*/*v*) for 2 h, followed by centrifugation at 10,000× *g* for 10 min at 4 °C. The supernatant containing B-MVs@5-ASA was collected for subsequent analysis. For characterization, both B-MVs and B-MVs@5-ASA were subjected to transmission electron microscopy (TEM), hydrodynamic diameter measurement, and zeta potential analysis. For TEM imaging, 10 μL of each fixed vesicle suspension was applied to a film-coated copper grid. The grid was negatively stained with 2% phosphotungstic acid (pH 7.0) for 2 min at room temperature, blotted, and dried before imaging. Transmission electron microscopy was performed using a JEM-1400Plus microscope (JEOL, Tokyo, Japan). The hydrodynamic diameter and zeta potential of both vesicle suspensions were determined using a Zetasizer Nano ZS (Malvern Instruments Ltd., Malvern Panalytical, Malvern, UK). The content of lipoteichoic acid in B-MVs and B-MVs@5-ASA was detected using a commercial LTA ELISA kit (Wuhan Adanti Biotechnology Co., Ltd., Wuhan, China). Based on the encapsulation efficiency of 11.4%, the calculated initial drug load in 1 mg/mL B-MVs@5-ASA is 14.8 μg, which represents M_0_. After incubating B-MVs@5-ASA in simulated gastric and intestinal fluids at 37 °C for 4 h, the mixture was centrifuged at 10,000× *g* for 10 min. The supernatant was collected, and its UV absorption curve was measured. The 5-ASA content in the supernatant (Mt) was calculated using the UV standard curve of 5-ASA. The 5-ASA drug release rate (%) = (Mt/M_0_) × 100%.

### 2.5. PKH67 Labeling of B. longum-Derived Extracellular Vesicles

RAW 264.7 macrophages were seeded in 6-well plates at 6 × 10^5^ cells per well and cultured for 12 h to allow adherence. PKH67-labeled *B. longum*-derived membrane vesicles (B-MVs) and B-MVs@5-ASA, prepared after quenching the labeling reaction, were added to each well (200 μL), followed by 1.8 mL of complete medium. The cells were incubated at 37 °C for 24 h, and then stained with Hoechst 33342 for 10 min at room temperature in the dark. The staining solution was aspirated, and the cells were maintained in PBS for imaging. Fluorescence signals were observed using an EVOS M5000 inverted fluorescence microscope (Thermo Fisher Scientific, Waltham, MA, USA).

### 2.6. Assessment of B. longum Extracellular Vesicles Biodistribution In Vivo

Purified B-MVs and B-MVs@5-ASA were labeled with 100 μL of Cy5.5-NHS solution (50 μg/mL) and incubated overnight at 4 °C with gentle rotation. The reaction was quenched with 10% BSA, followed by centrifugation at 100,000× *g* and 4 °C for 90 min. The supernatant was discarded, and the pellet was washed three times with PBS to remove excess dye before being resuspended in PBS. The labeled vesicles were orally administered to mice by gavage (300 μL per mouse). At 4 h post-administration, their biodistribution in vivo was monitored using an IVIS Spectrum imaging system (PerkinElmer, Waltham, MA, USA).

### 2.7. Lactate Dehydrogenase (LDH) Cytotoxicity Assay

RAW 264.7 macrophages and Caco-2 cells were seeded in 96-well plates at 5 × 10^3^ cells per well and allowed to adhere for 12 h. The cells were treated with PBS, B-MVs (5 mg/mL), or B-MVs@5-ASA (5 mg/mL). Each well was adjusted to a final volume of 200 μL with serum-free medium. At 2, 4, 6, and 8 h, the culture supernatant was collected for LDH analysis. The LDH Release Assay kit was purchased from Beyotime Biotechnology Co., Ltd. (Shanghai, China). For each sample, 120 μL of supernatant was mixed with the LDH reaction reagent according to the manufacturer’s instructions and incubated at room temperature for 30 min. Absorbance at 490 nm was measured using a microplate reader, and LDH release was calculated as:

LDH release (%) = (Experimental LDH release OD_490_/Maximum LDH release OD_490_) × 100%

### 2.8. In Vitro Assessment of LPS-Induced NO Production

RAW 264.7 macrophages were seeded into 96-well plates at 5 × 10^3^ cells/well and allowed to adhere for 12 h at 37 °C. The medium was replaced with serum-free DMEM, and cells were serum-starved for 4 h. An inflammatory response was induced by treating cells with lipopolysaccharide (LPS, 500 ng/mL) for 16 h. The nitric oxide (NO) level in the culture supernatant was then quantified using a commercial NO assay kit (Beyotime, Shanghai, China), following the manufacturer’s instructions.

### 2.9. LPS-Induced Inflammation Model in RAW 264.7 Macrophage

RAW 264.7 macrophages were seeded into 6-well plates at a density of 1 × 10^6^ cells per well and allowed to adhere for 12 h at 37 °C. The medium was then replaced with serum-free DMEM, and the cells were serum-starved for 4 h. After starvation, the cells were assigned to four groups (three biological replicates per group): control (CN), LPS, B-MVs, and B-MVs@5-ASA. The CN group received 2 mL of complete culture medium. The LPS group was stimulated with 500 ng/mL LPS (2 mL per well). For the B-MVs and B-MVs@5-ASA groups, cells were treated with B-MVs (5 mg/mL, 40 μL) or B-MVs@5-ASA (5 mg/mL, 40 μL), respectively, and each well was supplemented with complete medium containing 500 ng/mL LPS to reach a final volume of 2 mL. After 16 h of incubation, the culture supernatants were collected, and then the levels of TNF-α, IL-4, IL-6, IL-10, and IL-1β in the supernatants were quantified using ELISA kits (Wuhan Adanti Biotechnology Co., Ltd., Wuhan, China) according to the manufacturer’s instructions.

### 2.10. ELISA Analysis

The concentrations of tumor necrosis factor-α (TNF-α) and interleukins (IL-4, IL-6, IL-10, and IL-1β) in cells, as well as the levels of myeloperoxidase (MPO), reactive oxygen species (ROS), TNF-α, IL-4, IL-6, IL-10, and IL-1β in mouse colon tissues, were quantified using commercial ELISA kits (Wuhan Adanti Biotechnology Co., Ltd., Wuhan, China). Colon samples were homogenized in ice-cold PBS (pH 7.4) and centrifuged at 3000× *g* for 20 min at 4 °C. The resulting supernatants were used for ELISA detection according to the manufacturer’s instructions. Absorbance at 450 nm was recorded using a microplate reader, and cytokine concentrations were calculated from standard curves.

### 2.11. DSS-Induced Colitis Model and Treatment Protocol

Female C57BL/6 mice (6 weeks old) were purchased from Jinan Pengyue Experimental Animal Breeding Co., Ltd. (License No.: SCXK (Lu) 2022-0006, Jinan, Shandong, China) and acclimated for 7 days under standard conditions. The animals were randomly assigned to five groups (n = 12 per group): control (CN), DSS, 5-ASA, B-MVs, and B-MVs@5-ASA. From day 1 to day 7, colitis was induced by administering 2.5% DSS in drinking water, while the control group received regular water. From day 4 to day 13, mice received a daily oral gavage of 200 μL of 5-ASA (1 mg/mL), B-MVs (5 mg/mL), or B-MVs@5-ASA (5 mg/mL).

At the end of the 14-day experimental period, fresh fecal samples were collected from each mouse for 16S rRNA sequencing. Colon length was measured as a macroscopic indicator of intestinal injury. The distal colon was divided into portions for subsequent analyses, including ELISA quantification of ROS, MPO, and inflammatory cytokines (TNF-α, IL-1β, IL-6, IL-4, and IL-10), histopathological evaluation by hematoxylin–eosin and Alcian blue staining, and immunofluorescence analysis of the tight junction proteins ZO-1 and claudin-1.

All animal procedures were approved by the Animal Use and Care Committee of Binzhou Medical University and conducted in accordance with institutional and national guidelines.

### 2.12. Histomorphological Evaluation

Colonic tissues were excised, photographed, and measured for length. A distal segment was fixed in 4% paraformaldehyde for 24 h, embedded in paraffin, and sectioned at 5 μm thickness. For histological assessment, the sections were deparaffinized in xylene, rehydrated through a graded ethanol series, and subjected to hematoxylin–eosin (H&E) staining to evaluate general tissue architecture and inflammatory injury. Additional sections were processed using Alcian blue staining to assess goblet cell abundance and mucin production. After dehydration and clearing, all stained sections were mounted with neutral resin and examined under a light microscope (Olympus, Tokyo, Japan) for histomorphological evaluation.

### 2.13. Immunofluorescence Assay

Colonic tissues were cryosectioned at a thickness of 10 μm. After blocking with goat serum at 37 °C for 1 h, the sections were incubated with primary antibodies against ZO-1 (1:100) and claudin-1 (1:1000) overnight at 4 °C. The sections were then washed with PBS and incubated with DyLight 488/549-conjugated goat anti-rabbit IgG for 2 h at room temperature in the dark. After additional PBS washes, nuclei were counterstained with DAPI for 5 min, and the sections were mounted using neutral resin. Fluorescence images were acquired using a Zeiss LSM880 laser scanning confocal microscope (Carl Zeiss, Oberkochen, Germany) and fluorescence intensity was quantified using ImageJ software version 1.54p (National Institutes of Health, Bethesda, MD, USA).

### 2.14. 16S rRNA Sequencing Analysis

Fecal samples were collected and immediately frozen in liquid nitrogen. Microbial genomic DNA was extracted, and the V3–V4 region of the 16S rRNA gene was amplified using specific primers (forward: 5′-ACTCCTACGGGGAGGCAGCAG-3′; reverse: 5′-GGACTACHVGGGGTWTCTAAT-3′). Purified PCR products were used for library construction, quality assessment, and high-throughput sequencing was performed on the Illumina NovaSeq 6000 platform (Illumina, San Diego, CA, USA).

Clean reads were processed in QIIME 2 for denoising, clustering, and taxonomic assignment based on the SILVA 138 database. Alpha diversity was evaluated using the Chao1, Observed Species, Shannon, and Simpson indices, while beta diversity was assessed by PCoA, PCA, and NMDS analyses. Taxonomic profiles were visualized using bar plots, heatmaps, and Krona charts. Group differences in community composition were examined using ANOSIM and Adonis tests. Differentially abundant taxa were identified with STAMP and LEfSe to determine potential microbial biomarkers.

### 2.15. Statistical Analysis

All quantitative data are presented as the mean ± SEM. Differences among groups were assessed using one-way ANOVA with post hoc multiple comparison tests. Statistical significance was defined as *p* < 0.05. Analyses were performed using GraphPad Prism 9.5.0. Microbiome statistical analyses, including alpha-diversity metrics, beta-diversity distance matrices, and differential abundance testing, were conducted using QIIME 2 (v2023.2).

## 3. Results

### 3.1. Characterization of B-MVs and B-MVs@5-ASA

**Figure 1:**
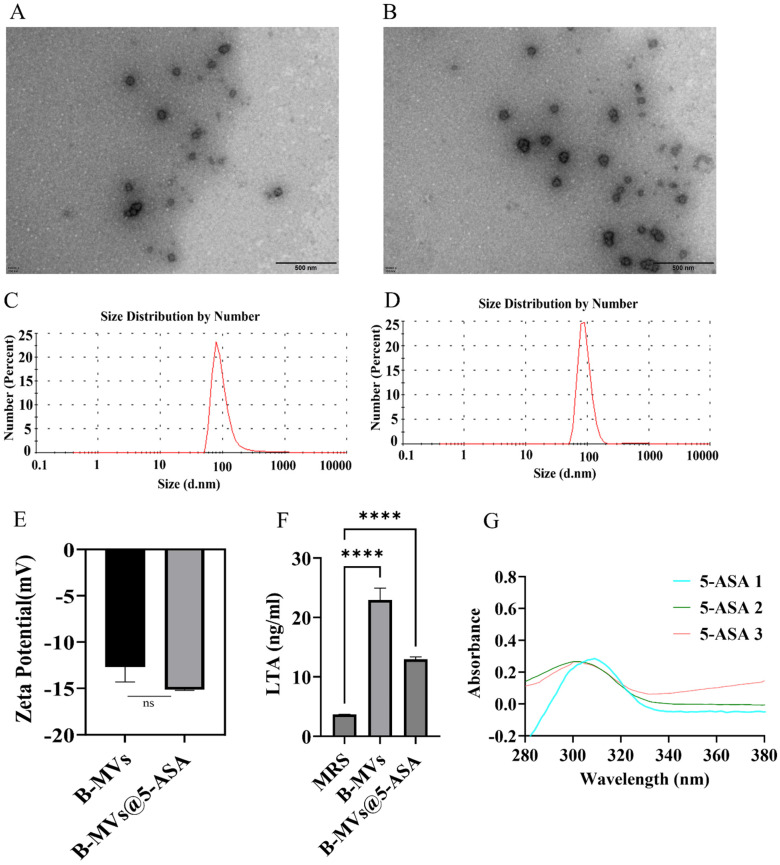
Characterization of extracellular vesicles (B-MVs) from *B. longum* and 5-ASA-loaded *B. longum* extracellular vesicles (B-MVs@5-ASA). (**A**) Transmission electron microscopy (TEM) image of native B-MVs. (**B**) TEM image of B-MVs@5-ASA showing preserved vesicle morphology after drug loading. (**C**,**D**) Size distribution profiles of B-MVs (**C**) and B-MVs@5-ASA (**D**) measured by dynamic light scattering. (**E**) Zeta potential analysis of B-MVs and B-MVs@5-ASA. (**F**) LTA levels in MRS medium, B-MVs, and B-MVs@5-ASA. (**G**) Quantification of unencapsulated 5-ASA in the supernatant used to calculate encapsulation efficiency. Data are presented as mean ± SEM. Statistical significance: ns “not significant”, **** *p* < 0.0001.

TEM analysis showed that native B-MVs displayed a spherical morphology (Figure 1A). A similar morphology was observed in B-MVs@5-ASA after drug loading, with no obvious aggregation or structural disruption in the TEM images (Figure 1B). Hydrodynamic diameter measurements revealed that B-MVs had a size of 63.72 ± 2.11 nm, which increased to 104.30 ± 2.81 nm in B-MVs@5-ASA, supporting the successful preparation of B-MVs@5-ASA (Figure 1C,D). The zeta potential of B-MVs@5-ASA changed slightly from −14.0 ± 0.82 mV to −15.23 ± 0.20 mV, with no significant difference (*p* > 0.05) relative to unloaded B-MVs, indicating 5-ASA loading did not affect surface charge (Figure 1E). As shown in Figure 1F, the MRS medium used as a negative control showed a very low LTA level (approximately 3.7 ng/mL). In contrast, the LTA content was markedly higher in the B-MVs group (approximately 22.9 ng/mL). Although the LTA level in the B-MVs@5-ASA group decreased to approximately 13.0 ng/mL after drug loading, it remained significantly higher than that in the MRS control group. A standard curve for 5-ASA quantification was generated using UV absorption spectra of 5-ASA solutions (5–60 μM; Figure S1A,B). By measuring unencapsulated 5-ASA in the supernatant, the encapsulation efficiency of 5-ASA in B-MVs was calculated as 11.14% ± 3.63% (Figure 1G). As shown in Figure S1C, the 5-ASA drug release rate from B-MVs@5-ASA after incubation in simulated gastrointestinal fluids for 4 h was calculated to be 55.7% ± 5.9%.

### 3.2. Cellular Uptake, In Vivo Distribution, Biocompatibility, and Anti-Inflammatory Activity of B-MVs and B-MVs@5-ASA

**Figure 2:**
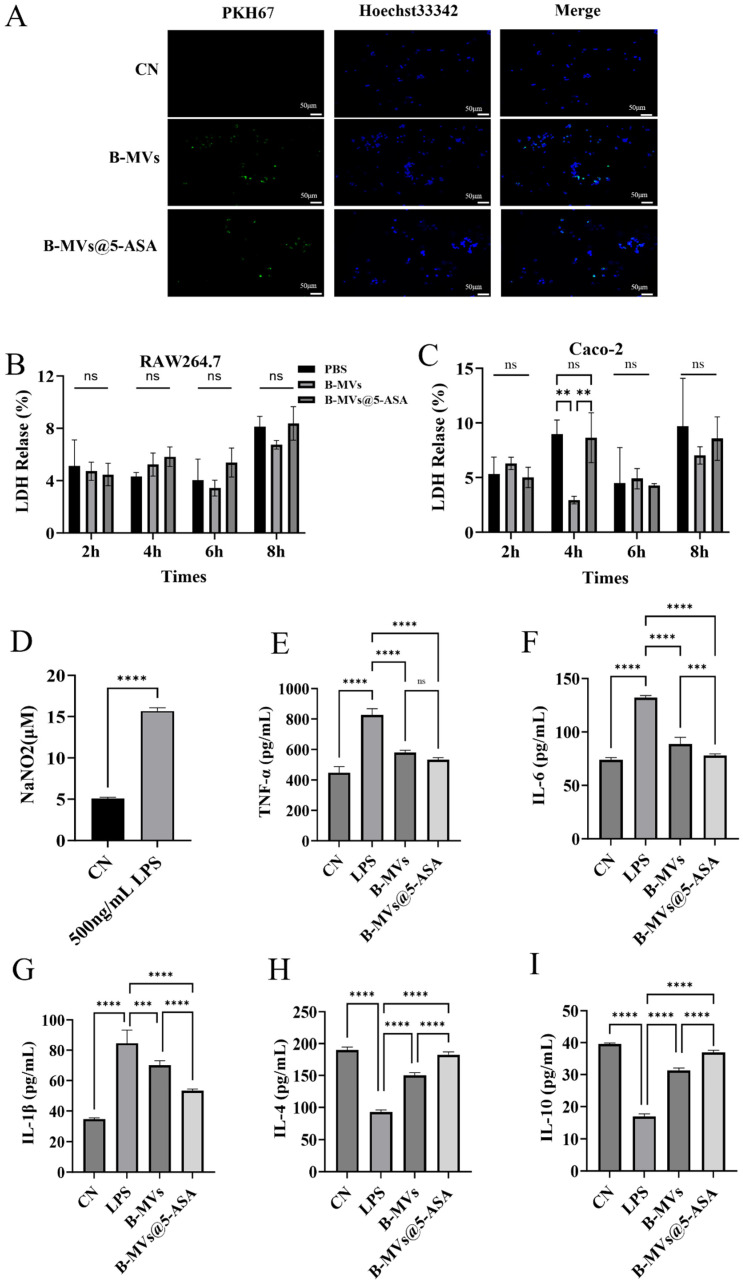
Cellular uptake, cytotoxicity, and inflammatory response associated with B-MVs and B-MVs@5-ASA. (**A**) Internalization of PKH67-labeled B-MVs or B-MVs@5-ASA (green) by RAW 264.7 macrophages, with nuclei counterstained using Hoechst 33342 (blue). (**B**) LDH release in RAW 264.7 cells treated with PBS, B-MVs, or B-MVs@5-ASA for 2, 4, 6, and 8 h (n = 3). (**C**) LDH release in Caco-2 cells treated with PBS, B-MVs, or B-MVs@5-ASA for 2, 4, 6, and 8 h (n = 3). (**D**) Establishment of an inflammatory model in RAW 264.7 cells following lipopolysaccharide (LPS) stimulation. (**E**–**I**) Levels of TNF-α, IL-6, IL-1β, IL-4, and IL-10 in culture supernatants measured by ELISA (n = 6). Data are presented as mean ± SEM. Statistical significance: ns “not significant”, ** *p* < 0.01; *** *p* < 0.001; **** *p* < 0.0001.

PKH67-labeled B-MVs and B-MVs@5-ASA were efficiently internalized by RAW 264.7 macrophages, as indicated by the colocalization of green vesicle signals with Hoechst-stained nuclei (Figure 2A). As shown in Figure S1D, IVIS images of mice at 4 h after oral gavage of B-MVs and B-MVs@5-ASA demonstrated distinct fluorescence patterns. The CN group exhibited only weak background signals, verifying the absence of autofluorescence. B-MVs group displayed diffuse or punctate fluorescence in the abdominal region, reflecting the distribution of bare vesicles. B-MVs@5-ASA group showed stronger and more concentrated signals in the colonic area. LDH assays showed no significant cytotoxicity in either RAW 264.7 or Caco-2 cells following treatment with B-MVs or B-MVs@5-ASA for up to 8 h (Figure 2B,C). As shown in Figure 2C, LDH release was significantly higher in the B-MVs group compared to the PBS and B-MVs@5-ASA groups at 4 h (*p* < 0.01). This likely results from a transient early interaction between the vesicles and cells. Importantly, this short-term difference does not affect the overall conclusion of good biocompatibility over the entire 8 h experiment.

LPS stimulation markedly increased NO production, confirming the successful establishment of the inflammatory model (Figure 2D). ELISA results showed that treatment with 5-ASA, B-MVs, or B-MVs@5-ASA significantly attenuated LPS-induced pro-inflammatory cytokine release. Among the three groups, B-MVs@5-ASA produced the most pronounced reductions, lowering TNF-α, IL-6, and IL-1β levels by 35%, 41%, and 37%, respectively (Figure 2E–G). In parallel, B-MVs@5-ASA elicited substantial increases in anti-inflammatory cytokines, with IL-4 and IL-10 elevated by 96% and 117.8%, respectively (Figure 2H,I). These quantitative results demonstrate that B-MVs@5-ASA exerts stronger anti-inflammatory activity than either 5-ASA or B-MVs alone.

### 3.3. Therapeutic Effects of B-MVs@5-ASA in DSS-Induced Colitis

**Figure 3:**
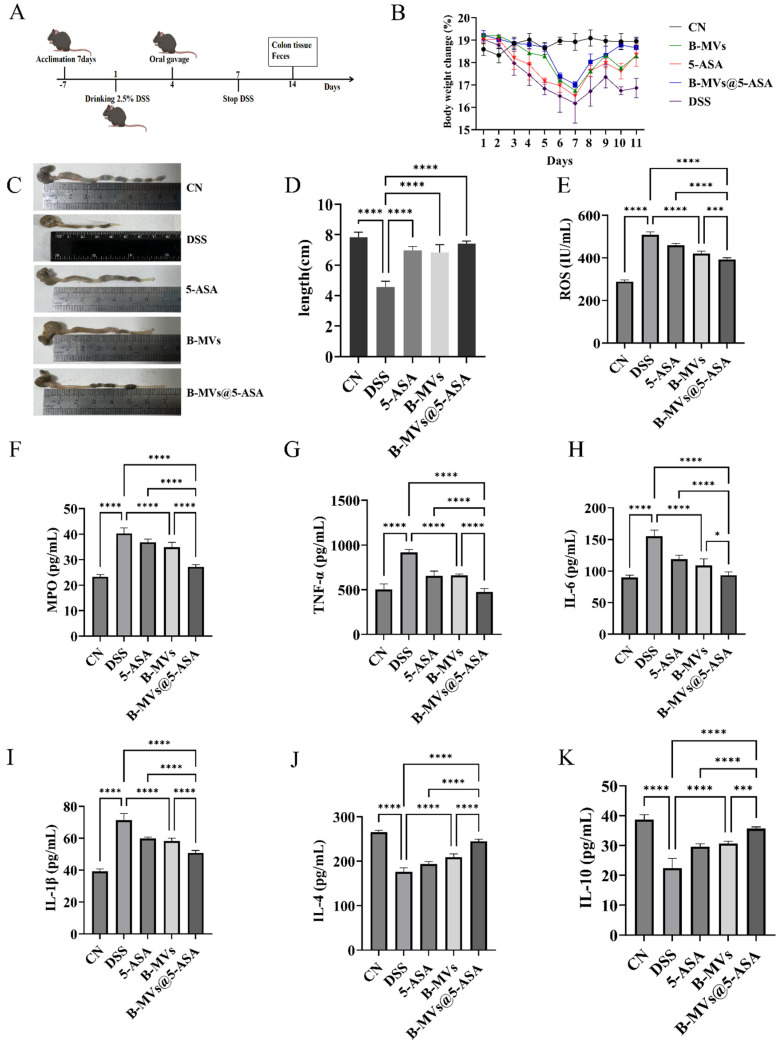
Therapeutic efficacy of B-MVs@5-ASA in a DSS-induced colitis mouse model. (**A**) Experimental workflow. Mice received sterile water or 2.5% DSS for 7 days. Oral administration of 5-ASA, B-MVs, or B-MVs@5-ASA began on day 4 and continued until the end of the study. Animals were sacrificed 7 days after DSS withdrawal. (**B**) Changes in body weight during the experimental period (n = 6). (**C**) Representative images of colons collected from mice in each group. (**D**) Quantification of colon length (n = 6). (**E**–**K**) Levels of ROS, MPO, TNF-α, IL-6, IL-1β, IL-4, and IL-10 in colon tissues measured by ELISA (n = 6). Data are presented as mean ± SEM. Statistical significance: * *p* < 0.05; *** *p* < 0.001; **** *p* < 0.0001.

DSS administration resulted in severe intestinal injury, as reflected by reduced colon length, elevated ROS and MPO levels, and strong induction of pro-inflammatory cytokines. All therapeutic interventions attenuated these abnormalities, with B-MVs@5-ASA showing the greatest degree of improvement among the treatment groups (Figure 3).

DSS treatment caused a clear reduction in body weight compared with the control group. All therapeutic interventions alleviated this weight loss, among which B-MVs@5-ASA showed the greatest improvement, with body weight recovering more effectively than both 5-ASA and B-MVs alone (Figure 3B). Colon length was restored to 6.97 cm by 5-ASA and 6.83 cm by B-MVs, whereas B-MVs@5-ASA achieved 7.40 cm, corresponding to a 62.6% recovery relative to DSS and the closest value to the normal control (*p* < 0.0001) (Figure 3C,D). Oxidative stress also showed a stepwise improvement across treatments: ROS decreased from 508.8 IU/mL (DSS) to 458.96 IU/mL (5-ASA), 420.57 IU/mL (B-MVs), and 392.26 IU/mL (B-MVs@5-ASA), with the latter showing the greatest reduction (*p* < 0.001) (Figure 3E). A similar trend was observed for MPO, which decreased modestly with 5-ASA and B-MVs but most substantially with B-MVs@5-ASA (27.23 pg/mL vs. 40.34 pg/mL in DSS) (*p* < 0.0001) (Figure 3F). DSS markedly elevated TNF-α, IL-6, and IL-1β, whereas B-MVs@5-ASA reduced these cytokines more effectively than either monotherapy (*p* < 0.05) (Figure 3G–I). TNF-α decreased from 919.87 pg/mL (DSS) to 658.35 pg/mL (5-ASA) and 661.37 pg/mL (B-MVs), but reached the lowest level with B-MVs@5-ASA (476.88) (*p* < 0.0001) (Figure 3G). IL-6 and IL-1β exhibited the same pattern of improvement. Anti-inflammatory cytokines were also more strongly enhanced by B-MVs@5-ASA than by the individual treatments. IL-4 increased from 176.44 pg/mL (DSS) to 193.27 pg/mL (5-ASA) and 208.78 pg/mL (B-MVs), reaching the highest level with B-MVs@5-ASA (244.77 pg/mL) (*p* < 0.0001) (Figure 3J). IL-10 followed a similar trend, with B-MVs@5-ASA producing the most pronounced increase (35.63 pg/mL vs. 22.41 pg/mL in DSS) (*p* < 0.001) (Figure 3K).

### 3.4. Restoration of Colonic Epithelial Barrier Integrity

**Figure 4:**
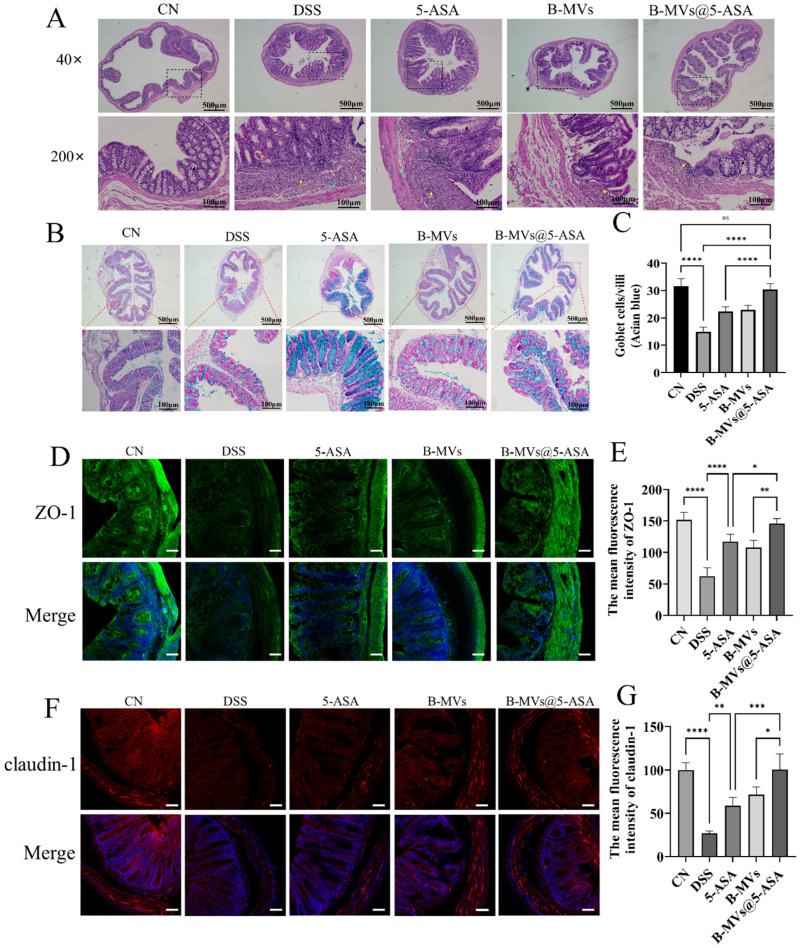
Assessment of colonic epithelial barrier integrity. (**A**) Representative H&E-stained colon sections from different treatment groups. Red arrows indicate epithelial integrity, black arrows indicate goblet cells, yellow arrows indicate inflammatory infiltration and fibrous hyperplasia, and blue arrows indicate submucosal inflammatory infiltration. (**B**) Representative Alcian blue staining showing goblet cell distribution in the colon. (**C**) Quantification of goblet cells per crypt (n = 5). (**D**) Immunofluorescence staining of ZO-1 (green); nuclei were counterstained with DAPI (blue). Scale bar: 50 μm. (**E**) Quantification of ZO-1 fluorescence intensity (n = 4). (**F**) Immunofluorescence staining of Claudin-1 (red); nuclei were counterstained with DAPI (blue). Scale bar: 50 μm. (**G**) Quantification of Claudin-1 fluorescence intensity (n = 4). Data are presented as mean ± SEM. Statistical significance: * *p* < 0.05; ** *p* < 0.01; *** *p* < 0.001; **** *p* < 0.0001.

H&E staining revealed pronounced epithelial injury in DSS-treated mice, including mucosal ulceration, crypt loss, and extensive inflammatory infiltration (Figure 4A). All therapeutic interventions attenuated these pathological changes, with 5-ASA and B-MVs showing partial restoration of epithelial structure. Notably, B-MVs@5-ASA produced the most substantial improvement, with markedly reduced ulceration, restored crypt architecture, and reduced inflammatory infiltration approaching that of the control group.

Alcian blue staining showed a significant reduction in goblet cells in DSS mice, indicating severe disruption of the mucus barrier (Figure 4B). Both 5-ASA and B-MVs moderately increased goblet cell numbers; however, B-MVs@5-ASA induced the most robust recovery, with goblet cell counts significantly higher than the DSS group (*p* < 0.0001) and nearly returning to control levels (Figure 4C).

Immunofluorescence analysis further demonstrated that DSS markedly reduced the expression of the tight junction proteins ZO-1 and claudin-1 (Figure 4D–G). All treatments enhanced the expression of these proteins, but B-MVs@5-ASA achieved the greatest restoration, increasing ZO-1 by 1.34-fold and claudin-1 by 2.73-fold relative to DSS mice. In addition, the membrane localization of both proteins was more continuous and structurally intact in the B-MVs@5-ASA group compared with the other intervention groups.

### 3.5. Modulation of Gut Microbiota Composition by B-MVs@5-ASA

**Figure 5:**
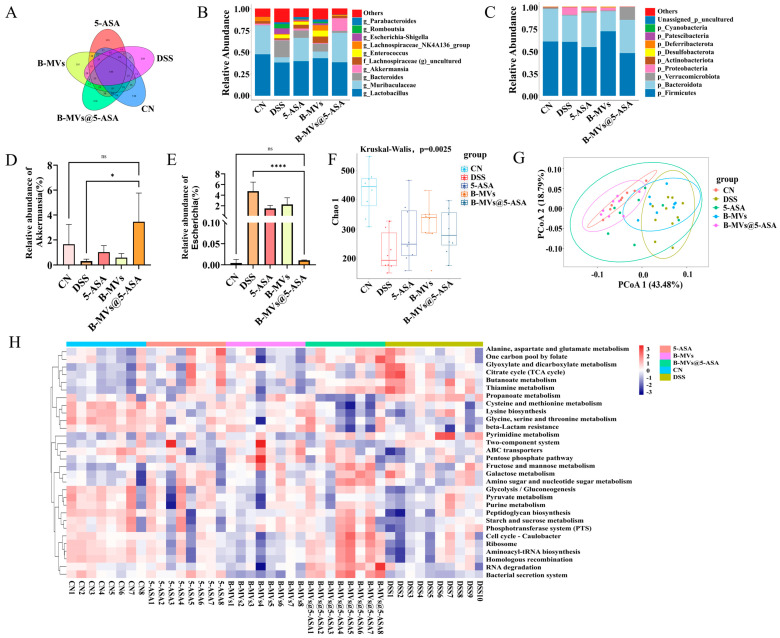
Effects of B-MVs@5-ASA on gut microbiota composition. (**A**) Venn diagram showing the number of unique and shared features among the different groups, based on 16S rRNA gene sequencing. (**B**) Relative abundance of dominant bacterial phyla in each group. (**C**) Relative abundance of dominant bacterial genera in each group. (**D**) Relative abundance of Akkermansia across groups (n = 3). (**E**) Relative abundance of Escherichia across groups (n = 3). (**F**) Chao1 richness index of the gut microbiota in each group. (**G**) Principal coordinates analysis (PCoA) based on unweighted UniFrac distances, illustrating differences in microbial community structure among groups. (**H**) KEGG-predicted functional pathway differences between the DSS and B-MVs@5-ASA groups. Data are presented as mean ± SEM. Statistical significance: ns “not significant”; * *p* < 0.05; **** *p* < 0.0001.

16S rRNA sequencing revealed significant alterations in microbial richness and community composition following DSS exposure. Observed features decreased from 730 in controls to 330 in DSS mice, whereas 5-ASA, B-MVs, and B-MVs@5-ASA partially restored richness, with B-MVs@5-ASA showing the highest recovery (418 features) (Figure 5A). At the phylum and genus levels, DSS reduced the abundance of Bacteroidetes and Verrucomicrobia and increased Proteobacteria, whereas all treatments reversed these trends, with the most pronounced restoration seen in the B-MVs@5-ASA group (Figure 5B,C). *Akkermansia* increased from 1.65% in DSS mice to 3.46% following B-MVs@5-ASA treatment (Figure 5D). In contrast, *Escherichia* rose to 4.75% after DSS exposure but declined to 2.25% in the B-MVs group, 1.50% in the 5-ASA group, and 0.01% in the B-MVs@5-ASA group (Figure 5E).

Analysis of the Chao1 index confirmed reduced microbial richness in DSS-treated mice, whereas all three treatments promoted restoration, with the highest richness observed in the B-MVs@5-ASA group (Figure 5F). β-diversity analysis via PCoA revealed distinct clustering among groups, with the B-MVs@5-ASA microbiota profile most closely resembling that of control mice (Figure 5G).

KEGG functional prediction showed that DSS induced marked shifts in microbial metabolic potentials. Treatment with 5-ASA, B-MVs, and particularly B-MVs@5-ASA partially corrected these DSS-induced functional alterations. The B-MVs@5-ASA group exhibited predicted functional profiles most similar to those of control mice (Figure 5H).

## 4. Discussion

*B. longum* is a key commensal bacterium that contributes to intestinal microecological balance and mucosal immune regulation [13,14]. Its extracellular vesicles (B-MVs) have been shown to mediate host–microbe communication and exert anti-inflammatory effects, partly through the induction of IL-10 [15]. Despite these biological advantages, the potential of BL-derived vesicles as therapeutic carriers remains insufficiently explored. In this context, our study investigates whether B-MVs can serve as a natural nanocarrier to enhance the delivery and bioactivity of 5-aminosalicylic acid (5-ASA), thereby expanding the therapeutic applicability of probiotic-derived vesicles in inflammatory bowel disease (IBD).

Biocompatibility is a fundamental requirement for the development of any oral nanocarrier-based drug delivery system [16]. In this study, the LDH assay demonstrated that neither RAW 264.7 macrophages nor Caco-2 epithelial cells exhibited significant LDH release following exposure to B-MVs@5-ASA, indicating minimal membrane damage and excellent biocompatibility (Figure 2A–C). RAW 264.7 cells, which participate in innate immune responses, and Caco-2 cells, a well-established model of intestinal epithelial integrity, together provide a representative evaluation framework for early toxicity screening [17,18]. Furthermore, in an LPS-induced RAW 264.7 inflammatory model, B-MVs@5-ASA effectively attenuated macrophage activation, as evidenced by reduced secretion of TNF-α, IL-1β, and IL-6, accompanied by a concomitant increase in IL-4 and IL-10 (Figure 2D–I). These results support the notion that B-MVs provide a non-toxic and immunomodulatory platform capable of enhancing the anti-inflammatory performance of 5-ASA. Notably, similar anti-inflammatory activities have been reported for probiotic-derived extracellular vesicles, including those from *B. longum* and *Lactobacillus plantarum* [6,19]. In parallel, nanocarrier-based 5-ASA delivery systems have been developed to improve colonic targeting and therapeutic efficacy [20]. More recently, probiotic EVs have been evaluated in combination with reduced-dose 5-ASA for ulcerative colitis, further supporting the therapeutic potential of integrating EV-mediated immunomodulation with 5-ASA treatment [21]. In this context, our study extends these previous efforts by incorporating both features into a single system, suggesting that BL-derived extracellular vesicles may serve not only as a biocompatible carrier for 5-ASA, but also as a delivery platform with potential immunomodulatory advantages.

The intestinal barrier is maintained by the coordinated integrity of the epithelial layer, goblet cell-derived mucus, and tight junction complexes [22,23]. DSS exposure disrupted all three components, as indicated by crypt distortion, substantial goblet cell depletion, and pronounced inflammatory infiltration. Treatment with B-MVs@5-ASA alleviated these pathological abnormalities, restoring epithelial continuity and mucus-producing cell numbers. These histological improvements were accompanied by restoration of tight junction integrity, as reflected by increased expression and appropriate membrane localization of ZO-1 and claudin-1 (Figure 4). Restoration of tight junction proteins is widely recognized as a key determinant of mucosal sealing and barrier resilience under inflammatory stress [24,25]. The DSS-induced increases in MPO and ROS were also markedly reduced following treatment (Figure 3E,F), suggesting attenuation of neutrophil-driven inflammation and oxidative stress [26,27]. Concomitantly, the inflammatory milieu shifted toward a state more favorable for epithelial repair. DSS-induced elevations in TNF-α, IL-1β and IL-6 were reduced following treatment, whereas IL-10 and IL-4 were increased (Figure 3G–K), suggesting that suppression of pro-inflammatory signaling may have contributed to epithelial recovery. Similar cytokine and barrier-protective effects have been reported for probiotic-derived extracellular vesicles, which promote mucosal healing in part by modulating epithelial-immune crosstalk [28,29]. Mechanistically, these effects may be partly attributable to the bioactive cargo of B-MVs. Previous studies have shown that *B. longum*-derived vesicles contain membrane-associated proteins involved in immune modulation and host interaction, including ABC transporters, quorum-sensing proteins, extracellular solute-binding proteins, and mucin-binding proteins [15,19]. In this context, B-MVs may contribute to epithelial protection and immune regulation not only by delivering 5-ASA, but also through the intrinsic bioactivity of their vesicular cargo. Taken together, these findings suggest that B-MVs@5-ASA reinforces barrier integrity during colitis through coordinated effects on epithelial repair, mucus preservation, tight junction restoration, and inflammatory regulation. These multimodal actions are consistent with growing evidence that microbial vesicles deliver bioactive molecules capable of strengthening epithelial junctions and limiting inflammatory injury [30], thereby supporting the therapeutic potential of this vesicle-based formulation in colitis.

Gut microbiota dysbiosis is widely recognized as a central contributor to the initiation and progression of inflammatory bowel disease, often characterized by reduced microbial diversity and enrichment of inflammation-associated taxa [31]. In the present study, B-MVs@5-ASA partially restored microbial richness and shifted community composition toward a profile more closely resembling healthy controls (Figure 5). Notably, the relative abundance of *Akkermansia muciniphila*, a bacterium associated with mucus layer maintenance and short-chain fatty acid production, increased markedly following treatment. Previous research indicates that *A. muciniphila* enhances mucus barrier thickness, reduces epithelial permeability, and attenuates inflammatory signaling [32], suggesting that its enrichment may contribute to the barrier-protective and anti-inflammatory effects observed. In addition to compositional improvements, predicted functional pathways provided further insight into microbial recovery. DSS exposure altered microbial metabolic functions, particularly those related to lipid processing and glycan biosynthesis, which are often disrupted under inflammatory stress [33]. Treatment with B-MVs@5-ASA shifted these pathways toward profiles associated with translation, energy metabolism, and signal transduction, indicating partial restoration of microbial functional capacity. Previous studies have shown that probiotic-derived extracellular vesicles can also remodel gut microbial communities during colitis. For example, vesicles derived from *L. plantarum* were reported to regulate intestinal microflora and associated metabolic pathways [6], whereas more recent work showed that *L. plantarum* EVs combined with reduced-dose 5-ASA improved gut microbial homeostasis and altered colitis-associated taxa in ulcerative colitis [21]. These reports are consistent with our findings that B-MVs@5-ASA partially restored microbial richness, reshaped community structure, and increased the abundance of *A. muciniphila*. Mechanistically, as mucosal inflammation subsides, mucus secretion is restored, and epithelial barrier integrity is re-established, the intestinal microenvironment may become more permissive for the re-establishment of beneficial microbial taxa and the recovery of microbial metabolic function. Collectively, these findings suggest that B-MVs@5-ASA modulates both microbial composition and functional potential, thereby contributing to the re-establishment of intestinal ecological homeostasis.

Several limitations of this study should be acknowledged. First, although B-MVs@5-ASA exhibited promising anti-inflammatory, barrier-protective, and microbiota-modulating effects, the molecular cargo of the vesicles was not systematically characterized, and the specific bioactive components responsible for these effects therefore remain undefined. Second, the microbiota analysis was based primarily on 16S rRNA sequencing and predictive functional profiling, without metabolomic validation, which limits our ability to directly link microbial compositional remodeling with metabolic recovery. Further studies integrating vesicle cargo characterization, metabolomic profiling, and targeted mechanistic analyses will be necessary to more fully elucidate how B-MVs@5-ASA exerts its therapeutic effects in colitis.

## 5. Conclusions

In summary, this study establishes extracellular vesicles derived from *B. longum* as an effective oral delivery platform for 5-ASA and demonstrates the therapeutic potential of the resulting B-MVs@5-ASA formulation in experimental colitis. The treatment ameliorated intestinal inflammation, reinforced epithelial barrier integrity, and contributed to the re-establishment of microbial ecological balance. These benefits were associated with reduced pro-inflammatory cytokine expression, enhanced anti-inflammatory signaling, and restoration of tight junction proteins such as ZO-1 and claudin-1. In parallel, shifts in microbial composition, including increased abundance of *Akkermansia* and decreased levels of pathogenic *Escherichia*, further supported mucosal recovery. Collectively, these findings highlight B-MVs@5-ASA as a promising probiotic vesicle-based therapeutic strategy for experimental colitis.

## Data Availability

The original contributions presented in this study are included in the article and Supplementary Material. Further inquiries can be directed to the corresponding authors.

## Supplementary Materials

The following supporting information can be downloaded at https://www.mdpi.com/article/10.3390/pharmaceutics18050553/s1: Figure S1. Characterization of 5-ASA and in vivo fluorescence imaging in mice.
