# Supplementary material for: Modulation of Inflammation and Gut Microbiota by a Bifidobacterium longum Extracellular Vesicle-Based Drug Delivery System for Alleviating Inflammatory Bowel Disease
Source: Pharmaceutics. 2026 Apr 30;18(5):553. doi: 10.3390/pharmaceutics18050553 (PMC13211112; doi:10.3390/pharmaceutics18050553)
Supplement: Supplementary file 1 [file pharmaceutics-18-00553-s001.zip › pharmaceutics-4213950-supplementary.pdf]

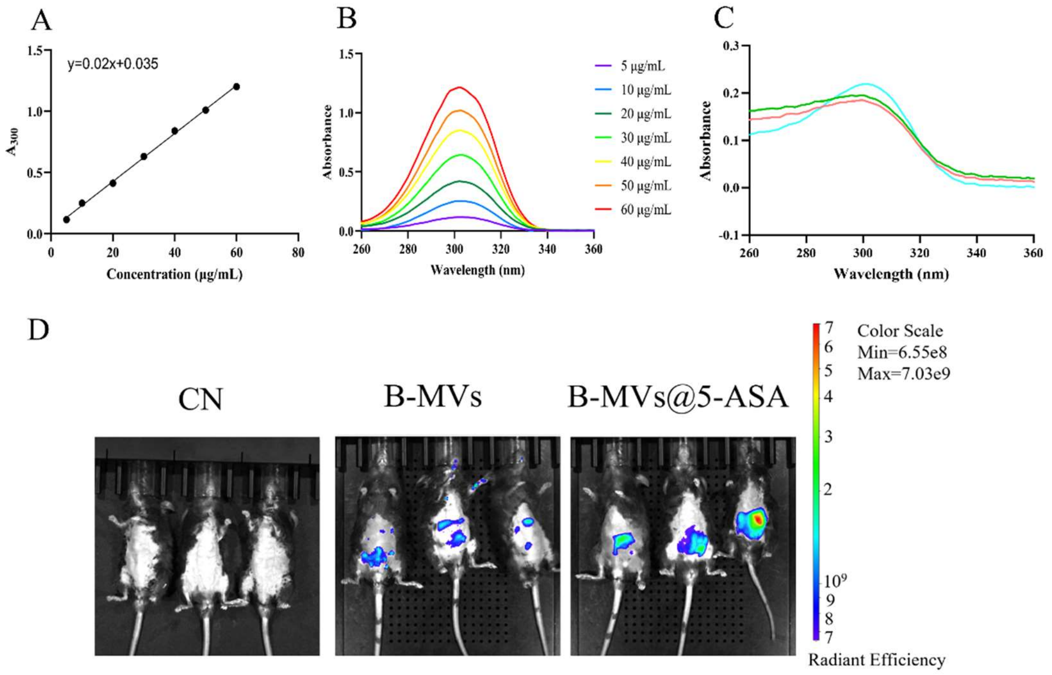

Supplementary Figure S1. Characterization of 5-ASA and in vivo fluorescence imaging in mice.

(A) Standard curve used for the quantification of 5-aminosalicylic acid (5-ASA). (B) UV-Vis absorption spectra of 5-ASA solutions at different concentrations. (C) UV-Vis spectra of 5-ASA released from B-MVs@5-ASA after 4 h incubation in simulated gastrointestinal fluids. (D) IVIS fluorescence images of mice at 4 h after oral gavage of CN, B-MVs, and B-MVs@5-ASA.
